# Examining EMTALA in the era of the patient protection and Affordable Care Act

**DOI:** 10.3934/publichealth.2018.4.366

**Published:** 2018-10-08

**Authors:** Ryan M. McKenna, Jonathan Purtle, Katherine L. Nelson, Dylan H. Roby, Marsha Regenstein, Alexander N. Ortega

**Affiliations:** 1 Department of Health Management and Policy, Dornsife School of Public Health, Drexel University, 3215 Market Street, Nesbitt Hall, Philadelphia, PA 19104, USA; 2 Department of Health Management and Policy, Dornsife School of Public Health, Drexel University, 3215 Market Street, Nesbitt Hall, Philadelphia, PA 19104, USA; 3 Department of Health Management and Policy, Dornsife School of Public Health, Drexel University, 3215 Market Street, Nesbitt Hall, Philadelphia, PA 19104, USA; 4 Department of Health Services Administration, School of Public Health, University of Maryland, 4200 Valley Dr # 2242, College Park, MD 20742, USA; 5 Department of Health Policy and Management, Milken Institute School of Public Health, George Washington University, 950 New Hampshire Ave NW, Washington, DC 20052, USA; 6 Department of Health Management and Policy, Dornsife School of Public Health, Drexel University, 3215 Market Street, Nesbitt Hall, Philadelphia, PA 19104, USA

**Keywords:** health policy, Affordable Care Act, emergency medicine, health reform, insurance reform

## Abstract

**Background:**

Little is known regarding the characteristics of hospitals that violate the Emergency Medical Treatment and Labor Act (EMTALA). This study addresses this gap by examining EMTALA settlements from violating hospitals and places these descriptive results within the current debate surrounding the Patient Protection and Affordable Care Act (ACA).

**Methods:**

We conducted a content analysis of all EMTALA Violations that resulted in civil monetary penalty settlements from 2002–2015 and created a dataset describing the nature of each settlement. These data were then matched with Thomson Healthcare hospital data. We then present descriptive statistics of each settlement over time, plot settlements by type of violation, and provide the geographic distribution of settlements.

**Results:**

Settlements resulting from EMTALA violations decreased from a high of 46 in 2002 to a low of 6 in 2015, a decline of 87%. Settlements resulting from violations most commonly occurred for failure to screen and failure to stabilize patients in need of emergency care. Settlements were most common in hospitals in the South (48%) and in urban areas (74%). Among Disproportionate Share Hospitals (DSH) with a violation, the majority (62%) were located in the South or in urban areas (65%). Violating hospitals incurred annual settlements of $31,734 on average, for a total $5,299,500 over the study period.

**Conclusions:**

EMTALA settlements declined prior to and after the implementation of the ACA and were most common in the South and in urban areas. EMTALA's status as an unfunded mandate, scheduled cuts to DSH payments and efforts to repeal the ACA threaten the financial viability of safety-net hospitals and could result in an increase of EMTALA violations. Policymakers should be cognizant of the interplay between the ACA and complementary laws, such as EMTALA, when considering changes to the law.

## 1. Introduction

Since its passage in 1986, the EMTALA has been one of the most comprehensive laws granting nondiscriminatory access to emergency medical care [22], [32]. EMTALA was originally conceived as a policy to prevent “patient dumping”, the refusal of EDs to treat patients who could not pay for treatment [22]. EMTALA mandates that a hospital must appropriately screen, stabilize, and, if necessary, transfer a patient regardless of insurance status or ability to pay. If it is deemed necessary that the patient needs to be transferred, they must be transferred to a facility with appropriate care and the receiving facility must accept the patient [31].

Federal enforcement of EMTALA is managed by two agencies, CMS and OIG. EMTALA investigations are initiated with a complaint being filed with one of the 10 regional CMS offices and typically submitted by patients, hospitals, or ED staff [1]. If a violation is confirmed by CMS field investigators, hospitals must submit a plan to correct deficiencies highlighted by CMS within 90 days [2]. Hospitals that fail to implement acceptable corrective actions risk termination of their Medicare provider agreements, which could result in a significant financial loss and lead to the closure of the facility. If the plan is accepted by CMS, the investigation ends; however, the OIG may still levy punitive fines on hospitals and physicians' offices. Fines have a maximum of $50,000 per hospital and physician and are not covered by physician malpractice insurance.

Since EMTALA's implementation, the rate of reported patient dumping has dropped substantially, with recent estimates from 2005–2014 showing rates as low as 1.7 violations for every 1,000,000 ED visits [27]. While these rates represent a sharp departure from previous highs in the 1980s, EMTALA violations suffer from underreporting and hospitals still face compliance issues [12]. Additionally, while EMTALA represents an important safety net for those without insurance coverage, it does not guarantee free care to the patient and is not intended as a substitute for routine care. Under EMTALA, patients cannot be denied emergency care based on inability to pay, but may still be billed after receiving care. This could result in bad debt for both the consumer (i.e., bankruptcy) and the provider (i.e., uncompensated care). EDs already provide more uncompensated care to the uninsured than hospitals or outpatient clinics combined and nationally this amounted to approximately $50 billion in 2013 (HHS.gov 2015).

The insurance expansion provisions of the 2010 ACA lowered the uninsured rate for individuals ages 18–64 years from a high of 18.4% in 2013 to 10.2% in 2016, a reduction of 45% [4]. While findings of the ACA's impact on ED utilization are mixed, some recent studies have shown that the ACA is associated with improvements in access to usual sources of care other than the ED and primary care utilization, especially for low-income groups and racial/ethnic minorities [3],[15],[25]. The expansion altered the payer-mix of many providers away from self-pay, which resulted in improved charge capture, reductions in uncompensated care, and potentially served to lower rates of patient dumping [7],[8]. Thus, after the national implementation of the ACA in 2014, we would expect to observe a decline in patient dumping and settlements arising from EMTALA violations. Several proposals to repeal and replace the ACA were estimated by the CBO to reverse nearly all of the gains in coverage attributable to the ACA [5],[6],[13]. Although those proposals ultimately failed to become law during the summer of 2017 legislative session, there are still proposals being circulated to reverse the ACA's insurance expansion through administrative action and by reducing Medicaid spending via the federal budget.

Hospitals serving large numbers of Medicaid and uninsured individuals are eligible for federal DSH payments, to help offset the costs of uncompensated care. Since the insurance expansion has likely worked to reduce the burden of uncompensated care, the ACA has built-in cuts to DSH payments to help reduce expenditures. These scheduled reductions to DSH payments will place greater strain on safety net hospitals. This increased strain could lead to patient dumping in order to avoid the increased shortfalls in revenue. The impact of DSH payments cuts will be magnified in Medicaid non-expansion states that have many low-income adults in the “coverage gap” [9],[18]. The uninsured low-income population are more likely than their privately insured counterparts to use EDs and impose a risk of uncompensated care for systems [14],[20].

In the face of reform efforts that would increase the number of uninsured patients and the scheduled cuts to DSH payments, it is important to understand the current prevalence of EMTALA violations and their distribution across the country. Despite its importance as a federal law mandating the provision of emergency medicine, little empirical work has been published on EMTALA violations and virtually none has examined the impact of the law within the context of the ACA and current debates about health care and insurance reform [1],[22],[26],[30].

This study adds to the current health reform debate by analyzing the content of all settled EMTALA violations from the OIG between 2002–2015 and identifying the prevalence and correlates of these cases. While settled fines do not constitute the universe of violations they are one of the few publically available markers by which to measure EMTALA violations. Additionally, while our findings are not causal in nature, we offer a descriptive analysis of these settlements and discuss the implications of results within the broader context of the current health care reform debate surrounding the ACA.

## 2. Methods

This study is a retrospective analysis of settled OIG civil monetary penalty settlements related to EMTALA violations from the OIG. Not every complaint which generates an EMTALA investigation results in a monetary settlement, thus we do not observe the entire universe of EMTALA complaints, only settled violations (approximately 7.9% of all violations [32]). In the event that a violation was found, CMS forwards the case to OIG, where the OIG decides if a monetary fine is warranted. We rely on settled cases as there are few reliable sources of data to assess patient dumping and cases settled by OIG have been used in prior work [30]. For brevity, we hereby refer to settlements that resulted from EMTALA violations as “settlements” in the manuscript.

The OIG website provides a one paragraph description about every EMTALA settlement since 2002. We conducted a content analysis of this information for all 191 settlements posted on the OIG website through 2015. A coding instrument was created in Qualtrics, a web-based survey tool, and each settlement was coded according to the nature of the violation and the characteristics of the patients involved [19]. We coded each settlement according to the year it occurred, the total dollar amount fined, the number of patients involved, and the type of violation that resulted in the settlement (i.e., failure to provide appropriate screening, failure to accept transfer, failure to provide appropriate transfer, failure to provide appropriate stabilization, or unknown). Settlements that occurred but had an unclear cause in the OIG reports were coded as “unknown” in our type of violation measure.

Hospital financial and geographic data from Thomson Healthcare Profile of US Hospitals were merged with the OIG EMTALA violation data by hospital name and address using Microsoft Excel 2016 [27]. The Thomson data include each hospital's unique Medicare ID, US Census region (Midwest, Northeast, South, West), geographic status (urban, rural), DSH status, and number of beds.

The merged dataset was imported into R [20] statistical software version 3.4.1 for analyses. First, annual trends in EMTALA settlements were plotted and stratified by type of violation which resulted in a settlement. Second, descriptive statistics were generated to describe hospital and geographic characteristics of violating hospitals. Third, 2010 Census data were used to calculate per-capita average fines at the state-level [30]. Results were plotted on a map of the US to visually explore geographic heterogeneity in EMTALA settlements and fines.

## 3. Results

We identified 191 EMTALA settlement agreements, which resulted in settlements that occurred between 2002–2015. A total of 24 hospitals could not be uniquely identified from the settlement reports or had active data in the Thompson database and were excluded from the analyses, for a total of 148 unique hospitals. After merging the settlements with the Thompson data, we had a sample of 167 settlements with associated hospital characteristics, none of which involved individual physicians.

**Figure 1.**
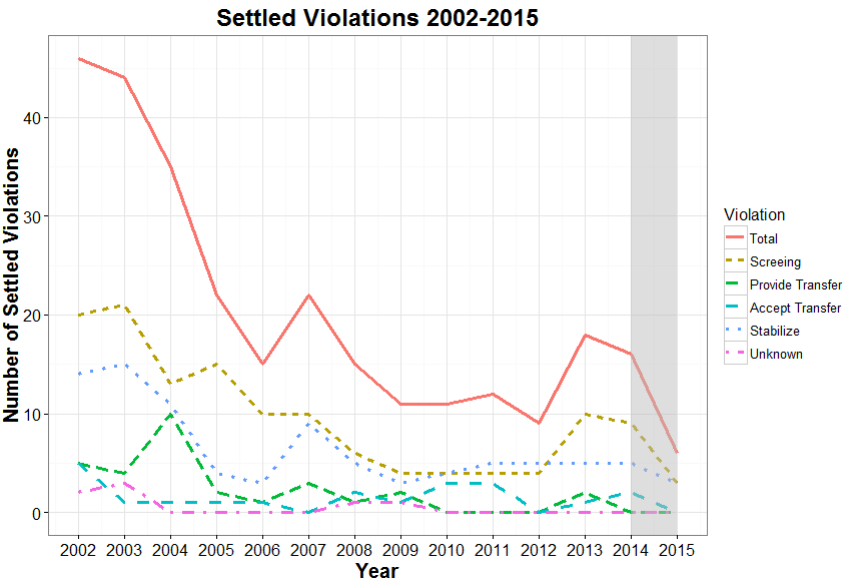
Settled EMTALA Violations by Type, Office of Inspector General 2002–2015. General (OIG) “patient dumping” settled violation case summaries from 2002–2015. These violations reflect settled cases, not all alleged EMTALA violations. The shaded bar represents the national implementation of the Patient Protection and Affordable Care Act.

Figure 1 shows annual trends in settled EMTALA violations from 2002–2015. The Figure is right skewed, which reflects a decline in the overall number of EMTALA settlements that range from a high of 46 in 2002 to a low of 6 in 2015, or a decline of 87%. Settlements did increase by 50% in 2013 relative to 2012, driven mostly by failure to appropriately screen. The shaded bar in Figure 1 indicates the national implementation of the ACA in 2014, which corresponds with a continued decline in overall settlements from 16 in 2014 to 6 in 2015. Throughout the 2002–2015 study period, the most common reason for a settlement was failure to appropriately screen followed by failure to stabilize.

Table 1 presents descriptive statistics of settled EMTALA settlements, as well as characteristics of violating hospitals. Nearly half of the settlements (47.9%) occurred at hospitals in the South, with the fewest settlements occurring in the Northeast (5.39%). Hospitals in urban areas (74.3%) were more likely than hospitals in rural areas (25.7%) to incur a settlement. Violating hospitals that had a settlement incurred an annual average fine of $31,734, for a total $5,299,500 over the study period. No hospital in the sample had its Medicaid provider agreement terminated as the result of an EMTALA settlement. Information on DSH status was unavailable in the Thomson data for 70 hospitals in the sample. For the hospitals for which their DSH status could be determined, sub analyses were conducted. Over a fifth of the hospitals (22.16%) had DSH status, with 62% of these hospitals located in the South and 65% located in urban areas.

**Figure 2.**
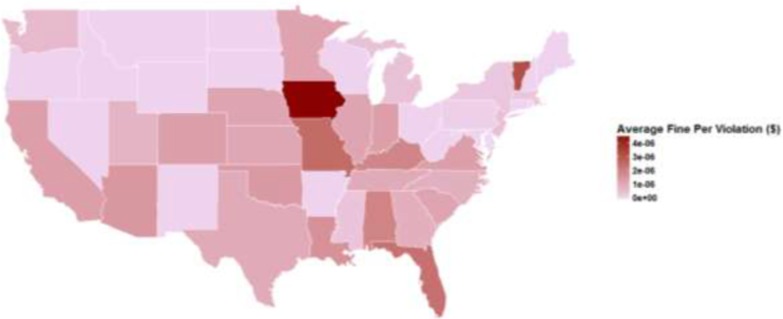
Per-capita Average Annual EMTALA Fines by State, 2002–2015. All fines are weighted by each state's population, as measured from the 2010 US Census.

Figure 2 displays distribution of average fines at the state-level adjusted by each state's population size. With the exceptions of Vermont and Iowa, per-capita fines are concentrated in the South and the Western US. As a robustness check, and to assess whether changes in the rate of EMTALA settlements might have resulted from differences in enforcement arising from changes to the OIG's budget, we calculated inflation-adjusted OIG budgets in from 2007–2015. We found that inflation-adjusted OIG budgets did not substantially decline over the 2007–2015 period and actually reached their highest levels in 2015.

**Table 1.** Descriptive Statistics of Settled EMTALA Violations, Office of Inspector General 2002–2015.

	2002	2003	2004	2005	2006	2007	2008	2009	2010	2011	2012	2013	2014	2015	Total
	(27)	(25)	(17)	(15)	(11)	(13)	(8)	(5)	(7)	(8)	(6)	(12)	(10)	(3)	(167)
Region															
Midwest	3.70	8.00	41.18	20.00	45.45	30.77	25.00	20.00	14.29	25.00	50.00	41.67	30.00	0.00	23.35
	(1)	(2)	(7)	(3)	(5)	(4)	(2)	(1)	(1)	(2)	(3)	(5)	(3)	(0)	(39)
Northeast	0.0	8.00	5.88	6.67	9.09	0.00	0.00	20.00	0.00	0.00	16.67	0.00	20.00	0.00	5.39
	(0)	(2)	(1)	(1)	(1)	(0)	(0)	(1)	(0)	(0)	(1)	(0)	(2)	(0)	(9)
South	48.15	52.00	41.18	46.67	27.27	38.46	62.50	40.00	71.43	62.50	33.33	58.33	40.00	66.67	47.90
	(13)	(13)	(7)	(7)	(3)	(5)	(5)	(2)	(5)	(5)	(2)	(7)	(4)	(2)	(80)
West	48.15	32.00	11.76	26.67	18.18	30.77	12.50	20.00	14.29	12.50	0.00	0.00	10.00	33.33	23.35
	(13)	(8)	(2)	(4)	(2)	(4)	(1)	(1)	(1)	(1)	(0)	(0)	(1)	(1)	(39)
Urban	66.67	76.00	58.82	80.00	81.82	84.62	75.00	100.00	100.00	75.00	66.67	66.67	70.00	66.67	74.25
	(18)	(19)	(10)	(12)	(9)	(11)	(6)	(5)	(7)	(6)	(4)	(8)	(7)	(2)	(124)
Violations*															
Screening	74.07	84.00	76.47	100.00	90.91	76.92	75.00	80.00	57.14	50.00	66.67	83.33	90.00	100.00	79.64 (133)
	(20)	(25)	(13)	(15)	(10)	(10)	(6)	(4)	(4)	(4)	(4)	(10)	(9)	(3)	
Accept Transfer	18.52	4.00	5.88	6.67	9.09	0.00	25.00	20.00	42.86	37.50	0.00	8.33	20.00	0.00	12.57
	(5)	(1)	(1)	(1)	(1)	(0)	(2)	(1)	(3)	(3)	(0)	(1)	(2)	(0)	(21)
Provide Transfer	18.52	16.00	58.82	13.33	9.09	23.08	12.50	40.00	0.00	0.00	0.00	16.67	0.00	0.00	17.96
	(5)	(4)	(10)	(2)	(1)	(3)	(1)	(2)	(0)	(0)	(0)	(2)	(0)	(0)	(30)
Stabilization	51.85	60.00	64.71	26.67	27.27	69.23	62.50	60.00	57.14	62.50	83.33	41.67	50.00	100.00	54.49
	(14)	(15)	(11)	(4)	(3)	(9)	(5)	(3)	(4)	(5)	(5)	(5)	(5)	(3)	(91)
Violation Not Clear	7.41	12.00	0.00	0.00	0.00	0.00	12.50	20.00	0.00	0.00	0.00	0.00	0.00	0.00	4.19
	(2)	(3)	(0)	(0)	(0)	(0)	(1)	(1)	(0)	(0)	(0)	(0)	(0)	(0)	(7)
Teaching Hospital	51.85	44.00	35.29	20.00	54.55	53.85	62.50	60.00	42.86	75.00	33.33	41.67	60.00	0.00	46.11
	(14)	(11)	(6)	(3)	(6)	(7)	(5)	(3)	(3)	(6)	(2)	(5)	(6)	(0)	(77)
DSH**	2.40	5.39	2.99	1.80	1.12	1.12	1.12	0.00	0.00	0.60	0.60	2.99	1.20	0.60	22.16
	(4)	(9)	(5)	(3)	(2)	(2)	(2)	(0)	(0)	(1)	(1)	(5)	(2)	(1)	(37)
Average Fine	23,815	23,300	25,882	29,800	40,000	22,788	34,688	59,000	37,714	39,438	43,833	36,666	42,075	58,333	31,733
	(27)	(25)	(17)	(15)	(11)	(13)	(8)	(5)	(7)	(8)	(6)	(12)	(10)	(3)	(167)
Mean Beds	299.81	299.81	311.65	201.20	310.73	286.62	440.38	257.40	389.29	490.13	331.00	353.50	216.70	233.00	302.54

Note: All values are averages, with counts shown in parentheses. These violations reflect settled cases, not all alleged EMTALA violations.

*Hospitals may have had more than one type of violation, thus the number of violations will be greater than the number of hospitals in the sample.

**Information on DSH status could not be obtained for 70 hospitals in the sample.

## 4. Discussion

In this study, we have observed a reduction in EMTALA settlements since 2002 and descriptively characterized that these settlements are most common among hospitals in the South and hospitals in urban areas. Since taking effect in 1986, EMTALA has been the only law granting universal access to emergency medical care regardless of ability to pay [22],[32]. While the ACA's insurance expansion significantly reduced the uninsured rate in the US, efforts to repeal the ACA threaten these reductions and could incentivize the utilization of EMTALA among the newly uninsured. In this study, we report trends in EMTALA settlements resulting in settlements and their geographic distribution before and after the national implementation of the ACA. Below, these findings are discussed within the context of the current health care reform debate to better understand the potential interplay between the ACA and complementary laws, such as EMTALA.

Over the study period, there was an overall strong downward trend in violations resulting in EMTALA settlements with the lowest number of settlements occurring after national implementation of the ACA. The overall downward trend in settlements prior to the ACA lends descriptive support to EMTALA's ability to reduce patient dumping. Furthermore, while a causal relationship cannot be determined from the present analysis, it is possible that the ACA helped to reduce patient dumping through two avenues, potentially even helping to reduce the observed spike in settlements occurring in 2013. First, in shifting hospitals' payer mix away from self-pay, the insurance expansion of the ACA reduces the risk of uncompensated care to systems. This reduced risk likely decreased the financial motivation for patient dumping. Second, the ACA helped improve access to health care at facilities other than the ED. While the findings of the relationship between ED utilization and the ACA are somewhat mixed, trends demonstrate that the ACA has helped to improve access to sources of care other than the ED [25],[26]. Furthermore, while our results are descriptive in nature, the OIG budget remained stable over this time, lending support to the notion that the observed decline was not due to a reduction in the OIG's enforcement capabilities. However, it is important to note that these results are only descriptive and not causal in nature.

Despite these improvements, settled violations resulting in EMTALA settlements and patient dumping still occur, albeit at a low incidence. Our results suggest that settlements resulting in settlements most frequently occur for failure to screen and stabilize patients. A prior study found a decrease in violations from nonclinical error over time, suggesting that the violations we observe from failure to screen and stabilize are reflective of true patient dumping [27]. We also find violations to be geographically concentrated in the Southern US and in urban areas. Settlements are likely more common in the South, as hospitals in Southern states in our sample were more likely to be disproportionate share hospitals in our observations.

While the ACA has been effective in reducing the burden of uncompensated care, several issues remain that might lead to hospitals dumping patients. First, if the ACA is repealed, the Congressional Budget Office projects that as many as 32 million US citizens could lose insurance coverage by 2026, the majority of which are low-income individuals [6]. EDs will likely bear a significant burden of uncompensated care resulting from the significant increase in the number of uninsured persons seeking care.

Second, scheduled cuts to DSH payments threaten the financial stability of safety-net hospitals, particularly those in non-expansion states [23]. Originally scheduled to take effect in 2014, the 2015 Medicare Access and CHIP Reauthorization Act delayed DSH cuts until fiscal year 2018. This delay prevented dependent hospitals from immediately foregoing reductions to the $11 billion used to cover uncompensated care, which is temporarily shielding hospitals from the reduction in revenue [7]. DSH payments will be reduced by $2 billion in 2018 and reduced by an additional $1 billion annually. Cuts will terminate in 2025 and will reach a maximum of $8 billion in both 2024 and 2025.

Given that DSH payments already fail to keep up with the rate of health care cost inflation, these cuts will serve to widen the DSH funding gap [16]. In the absence of federal or state assistance, shifting the financial burden of uncompensated care from the federal government to safety net hospitals could threaten the financial viability of these hospitals and potentially lead to patient dumping [16],[24]. Both an increase in the number of uninsured individuals and the pending cuts to DSH payments threaten the financial stability of hospitals, particularly safety-net hospitals. In response to increased financial strain and pressure, patient dumping may increase in EDs, specifically among the most financially vulnerable systems.

Patient dumping was once a huge problem and has the potential to resurface as the health care system goes through the shocks of health reform. Given the potential for an uptick in rates of patient dumping, it is paramount to continue to monitor EMTALA settlements. While settled cases represent one such metric to monitor the problem, information on both settled cases and complaints should be available to policy makers to gauge rates of patient dumping in the era of health reform.

This study has at least four main limitations. First, there is no uniform or transparent metric for measuring patient dumping. While we rely upon official OIG EMTALA settlement reports, it is likely that these underestimate the true incidence of settlements, as most complaints originate from hospital staff who may be reluctant to “blow the whistle” on a transferring hospital for fear of retribution. Second, we do not observe the universe of all EMTALA investigations. We only observe instances where a settlement occurred and resulted in a monetary fine by OIG. This prevents us from examining characteristics of hospitals that had investigations but were not found guilty of a violation, or which a monetary fine was not levied, which would be a useful comparison group to the violating hospital population. Additionally, this may result in a time lag between when the initial violation occurred and what we observe in our data, when the settlement was reached. However, these data still allow us to observe trends over time and these data have been used in comparable studies [28]. Third, our analyses are descriptive which precludes the ability to establish causation. Lastly, settlement data prior to 2002 were unavailable, and thus we are not able to examine trends in settlements since the inception of EMTALA in 1986.

## 5. Conclusions

Patient dumping once represented a substantial problem in emergent care, but has since been significantly reduced over recent decades. The insurance expansions of the ACA likely aided in this effort by shifting the payer-mix of many hospitals towards increased insurance coverage. This allowed for greater charge capture and likely worked to reduce patient dumping. However, while the frequency of patient dumping has declined, there is the potential for patient-dumping to reemerge as a problem. EMTALA's status as an unfunded mandate, the scheduled cuts to DSH payments, and the potential repeal of the ACA all threaten the financial viability of hospitals, specifically safety-net hospitals that received DSH payments. Shifting responsibility for uncompensated care from the federal government to local health systems could result in increased rates of patient dumping, reversing the observed downward trend in dumping over the past decade. In efforts to repeal the ACA, policymakers should be cognizant of the interplay between reform efforts and complementary laws, such as EMTALA. Reform efforts (e.g., Medicaid block grants or reductions in the generosity of insurance benefits or actuarial value) that significantly increase the number of uninsured individuals could have a deleterious impact on the financial stability of EDs, and could result in an increase in EMTALA violations.
